# Monomeric C-Reactive Protein Potential Utilization in the Histological Assessment of Inflammatory Bowel Disease (IBD) Patients

**DOI:** 10.7759/cureus.63200

**Published:** 2024-06-26

**Authors:** Simona Muresan, Mark Slevin, Emoke Szasz, Andrada Loghin

**Affiliations:** 1 Department of Internal Medicine, George Emil Palade University of Medicine, Pharmacy, Science, and Technology of Târgu Mureș, Târgu Mureș, ROU; 2 Center for Advanced Medical and Pharmaceutical Research, George Emil Palade University of Medicine, Pharmacy, Science, and Technology of Târgu Mureș, Târgu Mureș, ROU; 3 Department of Histology, George Emil Palade University of Medicine, Pharmacy, Science, and Technology of Târgu Mureș, Târgu Mureș, ROU

**Keywords:** inflammatory bowel disease, imunohistochemistry, crohn’s disease (cd), ulcerative colitis (uc), monomeric c-reactive protein

## Abstract

Introduction

Inflammatory bowel diseases (IBDs), including ulcerative colitis (UC) and Crohn’s disease (CD), represent chronic progressive inflammatory gastrointestinal disorders, without a single reference standard for their diagnosis. The histological assessment gained an important role in accurately measuring disease activity, and mucosal healing (MH) was recently proposed to be an ideal treatment goal for patients with IBD because of its favorable prognosis, with a lower risk of recurrence or surgical treatment. This paper aims to add to the histological classical findings for IBD patients the identification of the monomeric form of the C-reactive protein (mCRP) as a supplementary marker that could be stained at the level of tissue samples and could be correlated with the pathogenic mechanism.

Methods

Two groups of 10 patients were each selected for the study, for both UC and CD, together with a control group. All samples collected through digestive endoscopy were analyzed by using H&E-stained slides, followed by immunohistochemical examination with antibodies to mCRP (M8C10), and markers of inflammatory activity through CD3, CD45(leukocyte common antigen (LCA)), CD138/syndecan-1 and CD68.

Results

For the CD study group, all histological elements identified with H&E and afterward stained with CD138, CD68, CD3, and CD45/LCA were correlated with the standards imposed by the European Crohn’s and Colitis Organization (ECCO). For the group of patients with UC, histological images obtained with H&E and IHC stainings also confirmed the recommendation of ECCO. The main cells considered in the literature as histological markers for IBD are neutrophils, lymphocytes, and plasmocytes, stained in our study with CD45/LCA, CD3, and CD138. For all 20 cases of IBD (UC and CD), the staining with anti-Ab8C10 antibodies for mCRP was positive, while negative results were noticed within the control group. An mCRP protein visualized with anti-Ab8C10 antibodies presented an intracytoplasmatic localization in the neutrophils, plasma cells, lymphocytes, and macrophages from the lamina propria and glandular epithelium, without expression in endothelial cells.

Conclusions

Our study represents one of the first papers that identifies the localization of mCRP molecules within the intestinal mucosa of patients with IBD (both UC and CD) by using immunohistochemistry (IHC) staining. This finding opens a new perspective for considering mCRP as a marker correlated with histological disease activity and/or definition of histological remission in IBD.

## Introduction

Inflammatory bowel diseases (IBDs), including ulcerative colitis (UC) and Crohn’s disease (CD), represent chronic progressive inflammatory gastrointestinal disorders with a significant impact on quality of life and general well-being, causing bowel damage with frequent extraintestinal complications, hospitalizations, surgeries, and disability [1]. Until now, a single reference standard for the diagnosis of CD or UC does not exist. The diagnosis of CD or UC is based on a combination of clinical, biochemical, stool, endoscopic, cross-sectional imaging, and histological investigations [2].

The pathogenic mechanism in IBD has chronic inflammation as a main actor. The local and general overexpression of C-reactive protein (CRP), inflammatory cytokines, and adherent molecules contribute to vascular endothelial dysfunction, impaired fibrinolysis, activation of the coagulation cascade, and an abnormal platelet function, resulting in increased arterial and venous thrombosis with consequences at the level of intestinal mucosa [3,4]. The local inflammation alters the intestinal microbiota and the intestinal barrier, allowing toxic substances (and even inflammatory factors) produced by intestinal microorganisms to enter the circulatory system and induce extraintestinal complications [5].

In IBD, the mucosal lesions induced by chronic inflammation are explored by gastrointestinal endoscopy with biopsies. To date, no endoscopic feature is specific to CD or UC. The most useful endoscopic features of UC are continuous and confluent colonic involvement with clear demarcation of inflammation and rectal involvement. For CD, the most frequent endoscopic findings are discontinuous lesions, the presence of strictures and fistulae, and perianal involvement [2].

While in the last few decades, endoscopic remission has represented the most important treatment target, it was recently learned that up to one-third of patients with endoscopic healing may still have microscopic disease [6]. Consequently, the histological assessment gained a more important role in the accurate measuring of disease activity, and mucosal healing (MH), defined as histological remission, was recently proposed to be an ideal treatment goal for patients with IBD because of its favorable prognosis, with a lower risk of recurrence or surgical treatment [7].

There are typical microscopic features of IBD that are key factors for the diagnosis and definition of the level of histological activity that should always be reported by pathologists for a complete histological grading of disease activity. The main elements for the assessment of overall severity in UC are neutrophils in the lamina propria, in the surface epithelium, in the crypt epithelium (cryptitis), and within the lumen of crypts (crypt abscesses); basal plasmacytosis; lamina propria chronic inflammatory cell density; eosinophils in the lamina propria; erosions; ulcers; crypt architectural distortion; crypt atrophy; and mucin depletion [8]. For CD, the degree of architectural change or distortion, the degree of lamina propria chronic inflammation (lymphocytes and plasma cells), basal plasmacytosis, lamina propria and epithelial neutrophils, epithelial damage, granulomas, and erosions and ulcers are important indicative parameters [9]. Because of the heterogeneous histological information in IBD, a delay in formulating standardized scores and definitions of histological response, activity, and remission was recorded. Only two scores for histological inflammation in UC are fully validated: Robarts histopathology index (RHI) and Nancy index (NI) and that are still lacking for CD [8,10].

This paper aims to add to the histological classical findings for IBD patients the identification of a supplementary marker that, when characterized at the level of tissue samples, could be correlated with the pathogenetic mechanism. CRP is an acute-phase reactant that is widely accepted as a dominant serum biomarker in IBD trials [11]. Studies revealed that CRP could efficiently prognosticate patients in the active phase of IBD and, concomitantly, evaluate the efficiency of treatment through repeated measurement of this protein, but with limited accuracy in patients with lower activity [3]. When CRP meets activated cell and tissue components via the systemic circulation, the insoluble monomer of the native molecule: monomeric CRP (mCRP) is irreversibly produced in situ. Published literature from recent studies has already dictated the critical role of mCRP in the pathological initiation and progression of inflammatory diseases with vascular dysfunction, including atherosclerosis, neurodegenerative conditions such as Alzheimer’s disease (AD), and stroke [12]. Because the expression of mCRP within IBD has not been ascertained so far, the authors aimed to test its presence in biopsy samples using specific antibodies, to study their localization, and to correlate all results with standard histological findings validated in UC and CD.

## Materials and methods

Study groups and inclusion criteria 

A total of 20 patients examined by senior gastroenterologists between January 2018 and December 2023 were selected for this study. Demographic and pathological data were retrieved from institutional databases and repositories of County Hospital Târgu-Mureș, Romania, and original pathological reports from the Pathology Departments of the same medical institution.

These cases were organized into two study groups. The first group included ten patients with a confirmed diagnosis of UC, with clinical acute moderate relapse, for which rectosigmoidian biopsies were sampled. The second study group comprised ten patients with a diagnosis of CD and moderate clinical disease activity, with biopsies from the ileocecal area (six cases) and four patients with resection pieces after right hemicolectomy. A control group of 10 patients with biopsies without any pathology was also included in the study.

The H&E-stained slides from all the cases included in the study were reviewed by two pathologists (ES and AL) to confirm the diagnosis and classify the lesions into one of the study categories. For UC patients, histological activity was assessed with RHI [13]. For patients with CD, the main marker used for considering the histological activity was the neutrophilic inflammation of the epithelium, lamina propria, or both, according to the recommendations of the European Crohn’s and Colitis Organization (ECCO) [9].

Immunohistochemistry (IHC): technique and evaluation

For all study groups, the following IHC stainings were performed: antibodies to mCRP, CD3, CD45 (leukocyte common antigen (LCA)), CD138/syndecan-1, and CD68.

For the IHC exam of antibodies to mCRP, paraffin‐embedded tissue samples from 30 patients were processed and serial 5‐µm sections were cut. It used the avidin-biotin-peroxidase complex method (ABC Vectastain kit; Vector Laboratories, Peterborough, United Kingdom), and antibodies for mCRP (M8C10) were obtained and fully characterized as described previously by Schwedler et al. [14,15]. All antibodies were used at a dilution of 1:50. Paraffin‐embedded sections were deparaffinized, rehydrated, and boiled for 10 minutes in an antigen-unmasking solution of concentrated citric acid pH 6.0 as described by Slevin et al. [15,16]. Slides were incubated in a 0.5% v/v H2O2 in methanol for 30 minutes, with normal serum for 20 minutes, and then with a primary antibody (diluted in normal serum) for 30 minutes, followed by a 30‐minute incubation with biotinylated secondary antibody (diluted 1:50) and finally with ABC complex (diluted 1:50) for 30 minutes at room temperature. Staining was completed after incubation with 3,3′‐diaminobenzidine‐tetrachloride (DAB) substrate chromogen solution for three to ten minutes. Slides were counterstained with hematoxylin, dehydrated, cleared, and mounted in di‐n‐butyl‐phthalate‐polystyrene‐xylene (DPX) [15]. Negative control slides were performed in parallel, where the primary antibody was replaced with washing buffer and processed as above. No staining was seen in these sections (data not included).

For all other IHC examinations, formalin-fixed paraffin-embedded (FFPE) tissue sections were subjected to immunohistochemical staining, by using the following antibodies: anti-CD3, rabbit monoclonal primary antibody (clone 2GV6, Cat# 790-4341), incubated for 16 minutes; anti-CD45 (LCA), mouse monoclonal antibody (clone RP2/18, Cat# 760-2505), incubated for 16 minutes; anti-CD138/syndecan-1, mouse monoclonal antibody (clone B-A38, Cat# 760-4248), incubated for 32 minutes and anti-CD68, primary antibody (clone KP-1, Cat# 790-2931), incubated for 16 minutes. All staining procedures were performed on the Ventana BenchMark ULTRA (Ventana Medical Systems, Inc., Tucson, AZ) automated stainer using the UltraView DAB Detection Kit (Ventana, Cat# 760-500; Ventana Medical Systems, Inc., Tucson, AZ) following the manufacturer's protocols.

The examination of slides immunostained with M8C10 was performed in the Center for Advanced Medical and Pharmaceutical Research from George Emil Palade University of Medicine, Pharmacy, Science, and Technology of Targu Mureş (G.E. Palade UMPhST of Targu Mureș) and all H&E-stained slides together with the IHC exams for CD3, CD45 (LCA), CD138/syndecan-1, and CD68 were examined in the Department of Pathology, County Hospital of Targu Mureş.

All immunostaining results for all the cases were independently assessed by three pathologists (AL, ES, and MS). The controversial cases were evaluated in panels on a multiheaded microscope, and a consensus regarding the discordant features was reached. Detailed pathological reports were elaborated for each patient, with a clear correlation between the localization of each staining and different cell types.

The study was approved by both Ethical Committees from G.E. Palade UMPhST of Targu Mureș (document number 3145) and County Hospital Targu Mureș (document number 4416), and informed consent was obtained from all the patients included in this study.

## Results

Our series consists of 10 cases of patients with UC and 10 patients with CD for which both the H&E slides and IHC stainings were examined, together with a control group of 10 patients.

For the UC group, the average histological activity estimated on H&E slides and calculated with RHI was 18 out of 33 (min 11 and max 26). For CD patients, a minimum mild neutrophilic inflammation of the epithelium, lamina propria, or both were common findings in all cases included in our study.

The results of the immunohistochemical staining using single antibodies in both groups of patients are summarized in Table 1. It explains for each antibody, and both UC and CD study groups, the number of cases with weak immunostaining for a specific antibody (+), cases with moderate positive reactions (++), and cases with intense immunostaining (+++) for CD3, CD138, CD45/LA, CD68, or Ab8C10. There were no cases with negative reactions for each of the antibodies used for patients from the UC and CD study groups.

**Table 1:** Immunohistochemical results using single antibodies in patients with IBD +: weak positive reaction; ++: moderate positive reaction; +++: intense positive reaction; IBD: Inflammatory bowel diseases; LCA: leukocyte common antigen

IBD	Immunohistochemical markers				
	CD3	CD138	CD45/LCA	CD68	Ab8C10
Ulcerative colitis	+/++/+++ (0/3/7)	+/++/+++ (2/3/5)	+/++/+++ (0/6/4)	+/++/+++ (0/2/8)	+/++/+++ (2/4/4)
Crohn’s disease	+/++/+++ (0/4/6)	+/++/+++ (1/4/5)	+/++/+++ (0/4/6)	+/++/+++ (0/3/7)	+/++/+++ (0/3/7)

**Figure 1:**
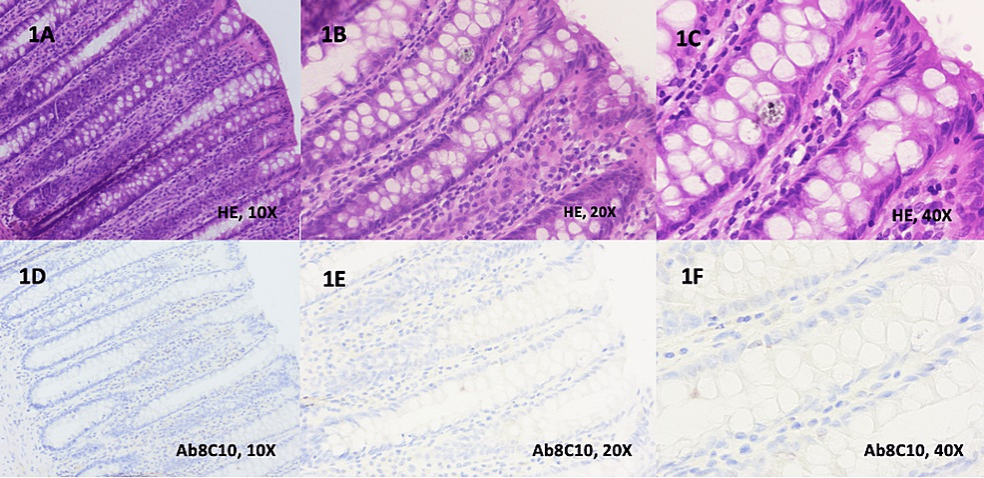
Immunohistochemical staining of individual anti-Ab8C10 antibodies for mCRP in the control group 1A. Colonic mucosa with normal histological aspect (H&E, 10x) 1B. Colonic mucosa with normal histological aspect (H&E, 20x) 1C. Colonic mucosa with normal histological aspect (H&E, 40x) 1D. Normal colonic mucosa with negative staining for anti-Ab8C10 antibodies (10x) 1E. Normal colonic mucosa with negative staining for anti-Ab8C10 antibodies (20x) 1F. Normal colonic mucosa with negative staining for anti-Ab8C10 antibodies (40x) (10x: images are magnified to a size 10 times larger than the original; 20x: images are magnified to a size 20 times larger than the original; 40x: images are magnified to a size 40 times larger than the original)

The main aim of this study was to identify mCRP at the level of the intestinal mucosa for patients with UC and CD and to clarify the localization of this protein correlated with specific features of IBD. Negative immunohistochemical staining of individual anti-Ab8C10 antibodies for mCRP was noticed within the control group, as it is shown in Figures 1A-1E.

**Figure 2:**
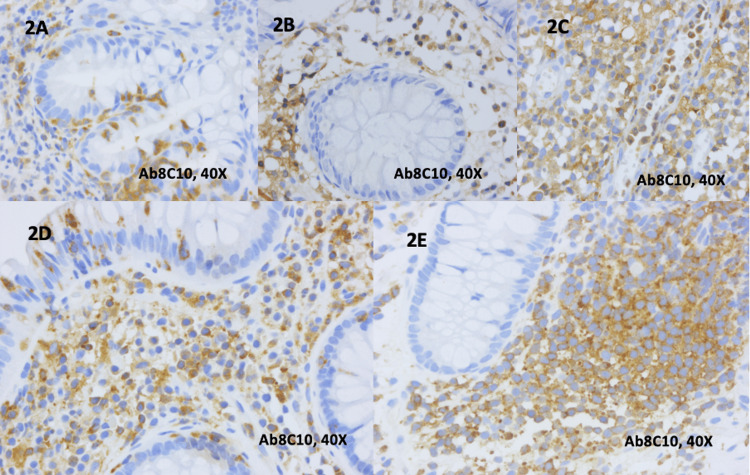
Immunohistochemical staining of individual anti-Ab8C10 antibodies for mCRP in ulcerative colitis and Crohn’s disease 2A. Histology of ulcerative colitis with activity features in the lamina propria. Note the presence of neutrophils, plasma cells, lymphocytes in the lamina propria and glandular epithelium, highlighted with anti-Ab8C10 antibodies (40x) 2B. Histology of ulcerative colitis with a mild increase in lymphocytes and plasma cells within the lamina propria immunostained with anti-Ab8C10 antibodies (40x) 2C. Histology of ulcerative colitis with an abundant chronic inflammatory infiltrate in the lamina propria. Note the presence of a blood vessel with inflammatory cells in the lumen immunostained with anti-Ab8C10 antibodies, without the staining of endothelial cells (40x) 2D. Histology of Crohn’s disease with neutrophils in the lamina propria and in the surface epithelium highlighted with anti-Ab8C10 antibodies (40x) 2E. Histology of Crohn’s disease with an abundant lympho-plasmocytic infiltrate in the lamina propria highlighted with anti-Ab8C10 antibodies (40x) (mCRP: monomeric C-reactive protein; 40x: images are magnified to a size 40 times larger than the original)

For all 20 cases of IBD (UC and CD), the staining with anti-Ab8C10 antibodies for mCRP was obtained. An mCRP stained using anti-Ab8C10 antibodies presented an intracytoplasmatic localization at the level of neutrophils, plasma cells, lymphocytes, and macrophages from the lamina propria and/or glandular epithelium, as is presented in Figures 2A-2E. For UC, mCRP was identified within intraluminal inflammatory cells from blood vessels, but without staining within endothelial cells or microvessels in these cases.

**Figure 3:**
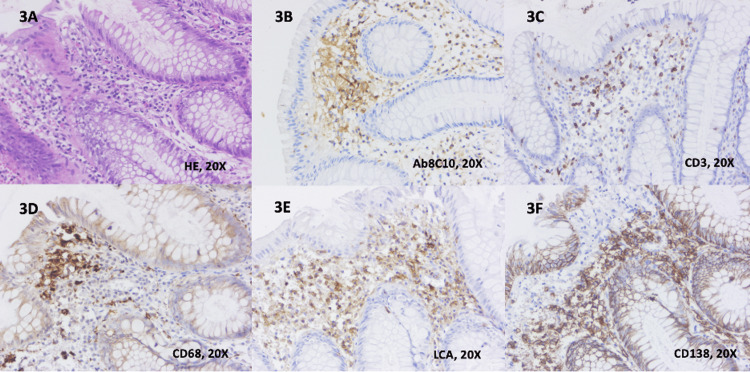
Histological findings and immunohistochemical staining in ulcerative colitis 3A. Colonic mucosa with normal glandular architecture and moderate chronic inflammatory infiltrate in the lamina propria (H&E, 20x) 3B. Positive cytoplasmatic staining of plasma cells, lymphocytes and macrophages with anti-Ab8C10 antibodies for mCRP (20x) 3C. Positive membrane and cytoplasmatic staining of lymphocytes with anti-CD3 antibodies (20x) 3D. Positive cytoplasmatic staining of the macrophages with anti-CD68 antibodies (20X) 4E. Positive cytoplasmatic staining of plasma cells and lymphocytes with anti-LCA antibodies (20x) 3F. Positive cytoplasmatic staining of plasma cells with anti-CD138 antibodies (20x) (mCRP: monomeric C-reactive protein; 20x: images are magnified to a size 20 times larger than the original; LCA: leukocyte common antigen)

Histological images from UC patients with mild clinical activity included neutrophils in the lamina propria and epithelium, cryptitis and/or crypt abscesses, plasmocytosis, and moderate chronic inflammatory infiltrate in the lamina propria. One of the cases is presented in Figures 3A-3F with both HE and IHC for Ab8C10, CD138, CD68, CD3, and CD45/LCA.

**Figure 4:**
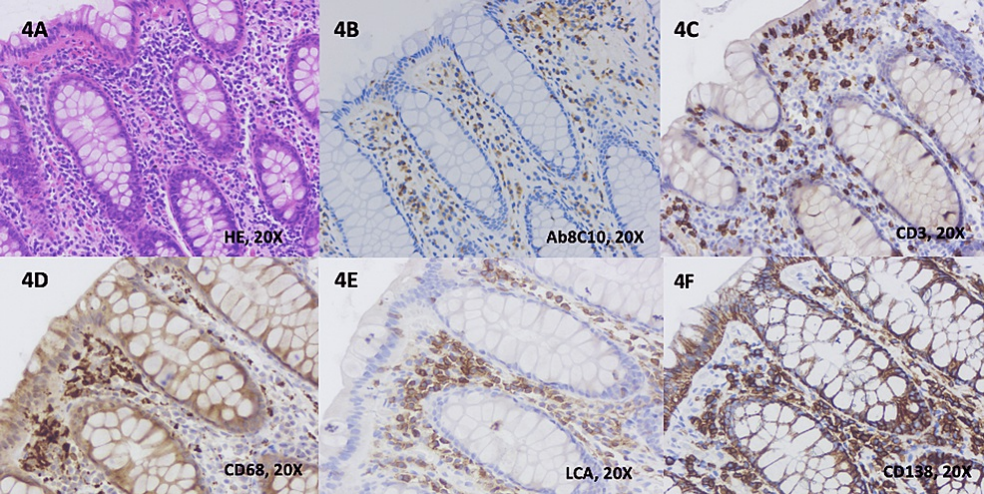
Histological findings and immunohistochemical staining in Crohn’s disease 4A. Colonic mucosa with a moderate chronic inflammatory infiltrate in the lamina propria and mild architectural distortions (H&E, 20x) 4B. Positive cytoplasmatic staining of plasma cells, lymphocytes, and macrophages with anti-Ab8C10 antibodies for mCRP (20x) 4C. Positive membrane and cytoplasmatic staining of lymphocytes with anti-CD3 antibodies (20x) 4D. Positive cytoplasmatic staining of the macrophages with anti-CD68 antibodies (20x) 4E. Positive cytoplasmatic staining of plasma cells and lymphocytes with anti-LCA antibodies (20x) 4F. Positive cytoplasmatic staining of plasma cells with anti-CD138 antibodies (20x) (mCRP: monomeric C-reactive protein; 20x: images are magnified to a size 20 times larger than the original; LCA: leukocyte common antigen)

For all patients with CD, we noticed a moderately abundant chronic inflammatory infiltrate of lamina propria (i.e., lymphocytes, macrophages, plasma cells), basal plasmacytosis, and the presence of neutrophils in the lamina propria and epithelium. Selected images from H&E slides and IHC staining of one patient with CD are included in Figures 4A-4F.

## Discussion

This retrospective study assessed the histological details of samples collected with digestive endoscopy from 20 patients with IBD. For the UC study group consisting of 10 patients, all histological elements identified with HE and afterward stained with CD138, CD68, CD3, and CD45/LCA were correlated with the standards imposed by ECCO [8]. For the group of patients with UC, histological images obtained with HE and IHC stainings confirmed the recommendation of ECCO as well [8,17]. The main cells considered within the literature as histological markers for IBD are neutrophils, lymphocytes, and plasmocytes, which were stained positively in our study (via anti-with CD138, CD3, and CD45/LCA, respectively). Additionally, CD68 was used to identify the presence of macrophages. These cells represent critical effector cells in the innate immune system and are essential for the regulation of intestinal homeostasis and inflammation particularly in the gut [18]. Zhang et al. emphasized the role of macrophages in the development of IBD, mainly the M1 phenotype, which is capable of secreting pro-inflammatory factors at the level of intestinal mucosa in patients with UC and CD [19].

Although the role of mCRP in IBD progression and development of extraintestinal complications was hypothesized within the literature [19], the identification of these molecules at the level of intestinal mucosa has not been examined in patients with UC and CD. Regarding the digestive system, there is a single study published by Kostner et al. that recently identified mCRP molecules in the tumoral areas of the colon [20]. Using artificial intelligence-based algorithms, the pattern of mCRP distribution was analyzed, and it was reported that the monomer was present exclusively within the tumor area, whereas adjacent normal colon mucosa showed no mCRP staining and that the level of expression strongly correlated with the level of circulating pentameric CRP (pCRP). Additionally, mCRP expression was significantly associated with tumor-infiltrating neutrophils, similar in that respect, to our study. The macrophages distributed in tumoral areas showed less mCRP positivity, compared with our results, suggesting that mCRP might just be an amplifier of the local inflammatory response in this case [20]. We consider that a correlation between the consistent data offered by this study with an extension of our study to a larger cohort of IBD patients could provide a novel mechanistic understanding of the risk of developing IBD-related neoplasia in patients with UC and CD.

The monomeric form of CRP has also been associated with cardiovascular pathology and was identified in atherosclerotic plaques and infarcted myocardium, where together with macrophages and complement factors is capable of inducing thrombus formation and excessive inflammation [21,22]. Studies performed in vitro showed that mCRP was expressed in neovessels of hypoxic cardiac tissues and intimal neovessels from complicated atherosclerotic lesions. Consequently, for cardiovascular diseases, mCRP is considered a regulator of signaling pathways associated with both angiogenesis and inflammation [23]. A large domain where mCRP was investigated is the nervous system, through in vivo and in vitro studies. Following a stroke, mCRP was identified in large quantities in peri-infarcted and infarcted brain tissue, particularly in the microvessels, being able to induce neurodegeneration and inflammation potentially perpetuating dementia [24,25].

In cardiovascular and neurovascular diseases and in the tumoral pathology of the colon, mCRP was stained in endothelial cells, supporting the hypothesis that these cells are targeted for the dissociation of pCRP into mCRP, with perhaps a critical contribution to the localization of the inflammatory response [20-25]. In our study, and in contrast, mCRP was expressed only in intravascular inflammatory cells, without association with vascular endothelium suggesting at least partially a nonsystemic source and likely in situ precipitation.

The strength of this study is the novelty of its theme. The identification and localization of mCRP molecules within intestinal mucosa in IBD patients are described here for the first time. Additionally, the correlation with previous studies in similar pathologies supports the hypothesis of CRP/mCRP as a potential key biomarker [11], while the presence of the dissociated monomer in the intestinal mucosa opens new directions for future therapeutical approaches that could positively influence the evolution of the intestinal inflammation and further extraintestinal complications, for example, by blocking localized CRP-mCRP dissociation.

The limitations of this study include, as a first factor, the size of the patient groups. Having the theoretical correlation between CRP-mCRP-intestinal mucosal inflammation-IBD as a starting point [3], we tested the hypothesis of the existence of mCRP in intestinal samples from CD and UC patients starting with 20 subjects. The positive results obtained until now impose a future extension of the study for a larger cohort. A multidisciplinary, well-designed, prospective study with a larger team of investigators is needed for such a desideratum. This approach could be finalized with the inclusion of mCRP within the list of standard histological markers that could be used for the assessment of histological activity in IBD. Finally, our samples, although whole slides, only represent isolated pictures of the immunological process for IBD patients and do not reflect the long-term evolution. Consequently, serial biopsies should be analyzed in dynamics to evaluate how mCRP impacts CD and UC evolution.

## Conclusions

Our study represents one of the first papers that identifies the localization of mCRP molecules within the intestinal mucosa of patients with IBD (both UC and CD) by IHC. This finding opens new perspectives for considering mCRP as a marker correlated with histological disease activity and/or the definition of histological remission and mucosal healing in IBD. Even though serum native (Hs)CRP cannot be used alone as a surrogate for histological activity in UC and CD, the monomeric form stained using an IHC technique could be included in future histological scores that should be validated in clinical studies for a possible prediction with increased accuracy of the relapse and hospitalization rates, the response to therapy, and the risk of developing IBD-related neoplasia for this category of patients.
